# YOLOv5-KCB: A New Method for Individual Pig Detection Using Optimized K-Means, CA Attention Mechanism and a Bi-Directional Feature Pyramid Network

**DOI:** 10.3390/s23115242

**Published:** 2023-05-31

**Authors:** Guangbo Li, Guolong Shi, Jun Jiao

**Affiliations:** 1 School of Information and Computer, Anhui Agricultural University, Hefei 230036, China; liguangbo@stu.ahau.edu.cn (G.L.); shigl@ahau.edu.cn (G.S.); 2 Key Laboratory of Agricultural Sensors, Ministry of Agriculture and Rural Affairs, Hefei 230036, China; 3 Anhui Provincial Key Laboratory of Smart Agricultural Technology and Equipment, Hefei 230036, China

**Keywords:** object detection, pig recognition, YOLOv5, attention mechanism, feature fusion

## Abstract

Individual identification of pigs is a critical component of intelligent pig farming. Traditional pig ear-tagging requires significant human resources and suffers from issues such as difficulty in recognition and low accuracy. This paper proposes the YOLOv5-KCB algorithm for non-invasive identification of individual pigs. Specifically, the algorithm utilizes two datasets—pig faces and pig necks—which are divided into nine categories. Following data augmentation, the total sample size was augmented to 19,680. The distance metric used for K-means clustering is changed from the original algorithm to 1-IOU, which improves the adaptability of the model’s target anchor boxes. Furthermore, the algorithm introduces SE, CBAM, and CA attention mechanisms, with the CA attention mechanism being selected for its superior performance in feature extraction. Finally, CARAFE, ASFF, and BiFPN are used for feature fusion, with BiFPN selected for its superior performance in improving the detection ability of the algorithm. The experimental results indicate that the YOLOv5-KCB algorithm achieved the highest accuracy rates in pig individual recognition, surpassing all other improved algorithms in average accuracy rate (IOU = 0.5). The accuracy rate of pig head and neck recognition was 98.4%, while the accuracy rate for pig face recognition was 95.1%, representing an improvement of 4.8% and 13.8% over the original YOLOv5 algorithm. Notably, the average accuracy rate of identifying pig head and neck was consistently higher than pig face recognition across all algorithms, with YOLOv5-KCB demonstrating an impressive 2.9% improvement. These results emphasize the potential for utilizing the YOLOv5-KCB algorithm for precise individual pig identification, facilitating subsequent intelligent management practices.

## 1. Introduction

With the progress of society and the rise in people’s income, the consumption of meat maintains an upward trend, which has resulted in a sharp surge in the demand for livestock, including live pigs, and prompted heightened expectations for the animals’ welfare and productivity [1,2,3]. A current topic of interest is finding an accurate and efficient solution to the problem of individual pig identification. Traditional methods such as painting, branding, ear tagging, and radio frequency identification (RFID) require significant human and material resources, resulting in a high workload and low efficiency. This is not conducive to intelligent [4,5,6], healthy, and precise pig farming [7,8]. Therefore, there is an urgent need for a simple and efficient method for individual pig identification.

Individual pig identification methods can be invasive or noninvasive. In recent years, farming has gradually adopted non-invasive methods, using neural networks as machine learning progresses. For instance, Wang Z et al. [9] proposed a two-stage pig face recognition method based on triple-edge loss. This approach ensures non-contact pig face recognition and significantly improves the average accuracy of pig face recognition to 94.04%. Similarly, an enhanced CNN model was developed by Hansen M F et al. [10] to facilitate the recognition of pig faces for non-invasive biometric identification purposes, achieving a certain level of improvement. Marsot M et al. [11] improved the algorithm for pig face recognition by detecting and refining the algorithms for pig face and pig eye, ultimately achieving an accuracy of 83%. In another study, Wang Z et al. [12] have introduced an enhanced ResNAM network as the principal framework for pig face image feature extraction, resulting in an improvement of 3% in recognition accuracy. Lastly, Posta, E et al. [13] have developed an algorithm specifically for instance segmentation of live pigs, achieving an accuracy of 91%.Additionally, Ahn H et al. [14] presents a novel method for detecting individual pigs, solving the problem of difficulty in detecting pigs due to high exposure, with an accuracy of up to 94.33%. Ocepek M et al. [15] proposed establishing an automated detection system for live pigs to improve recognition accuracy by optimizing the YOLOv4 algorithm, achieving up to 90% accuracy. Li, Y et al. [16] proposed an adaptive pig individual detection algorithm that introduces new parameters into the Gaussian mixture model and uses different learning rates to improve identification of live pigs in motion. Zhuang, Y et al. [17] suggested creating a pig feeding-and-drinking behavior software that identifies and judges pig behavior using convolutional neural networks, providing technical support for intelligent pig breeding. Yu S et al. [18] put forward a pig individual recognition method for edge devices that uses deep learning algorithms to detect pig individuals and pruning technology to improve computing efficiency. Seo J et al. [19] proposed a lightweight pig automatic detection network that reduces computational load through filtering and clustering modules, achieving 8.7 times higher performance than the original detector. Sa J et al. [20] suggested using image denoising and simple processing technologies to detect pig individuals, with an accuracy of 0.79 and an execution time of 8.71 ms. Cowton J et al. [21] developed a pig multi-object detection and tracking system using regional convolutional neural networks and real-time multi-object tracking. T. Psota et al. [22] proposed a tracking method based on probabilistic detection, assigning unique identifiers to instances using a classification network and assigning ear tags through algorithms, and creating a set of continuous trajectories for real-time tracking. Wang M et al. [23] optimized FairMOT with a deep learning algorithm, realizing the recognition and tracking of reappearing pig individuals and improving the accuracy of multi-target recognition and tracking. Bhujel A et al. [24] proposed a deep-learning-based pig individual and motion state detection and tracking algorithm and found that the YOLOv4 detector combined with the deep sorting tracking algorithm improves performance in pig multi-object detection and tracking. Zhang L et al. [25] proposed a method for individual detection and tracking of live pigs that achieves a recognition accuracy of 94.72% and a recall rate of 94.74%, providing appearance features for the tracker.

Despite the positive results achieved in various studies on pig identification, there are still certain limitations in practical pig farming, specifically with regards to identifying individual pigs from a distance. Evidently, most of these studies focus on pig faces, which are subject to occlusion and loss of features on the rear, left, and right sides during pig activities, leading to missed detections. To improve accuracy, this article proposes using pig heads and necks, which, taken together, contain more feature information for individual recognition. However, there is still room for improvement in terms of the robustness and accuracy of the algorithms mentioned above. Building upon the YOLOv5 algorithm, this article enhances the adaptability of the algorithm’s target frames, position feature extraction, and high-to-low-level feature fusion by optimizing target anchors, introducing CA attention mechanisms, and BiFPN feature fusion. These enhancements enable precise identification of individual pigs in practical farming settings.

The main contributions of this article are as follows:

(1) In terms of datasets, a new pig head and neck dataset has been created based on the pig face. Pig heads and necks are involved in the judgment criteria of pig recognition, which provides more information and improves accuracy.

(2) In terms of algorithms, this article intends to use the YOLOv5 algorithm, which has higher recognition accuracy, as the basis for improvement. Firstly, we will change the Euclidean distance of K-means clustering to 1-IOU to improve the adaptability of the algorithm’s target box. Then we will introduce coordinate attention mechanism to more effectively learn the features of small targets and target positions. Finally, we will improve the feature fusion method by introducing BiFPN feature fusion, which expands the algorithm’s receptive field and enhances multi-scale learning of multiple interfering targets. This will improve the accuracy of general pig individual identification, as well as the missed detection in dense pig scenes and the missed detection, false detection, and low confidence in classifying small targets at long distances.

(3) In terms of actual breeding, experiments have shown that the improved algorithm for pig recognition performs well in scenarios with single or multiple pigs, small targets, high density, and obstacles. This lays the foundation for intelligent, health-encouraging, and precise breeding of pigs.

## 2. Dataset and Methods

### 2.1. Dataset

#### 2.1.1. Dataset Acquisition

**Figure 1:**
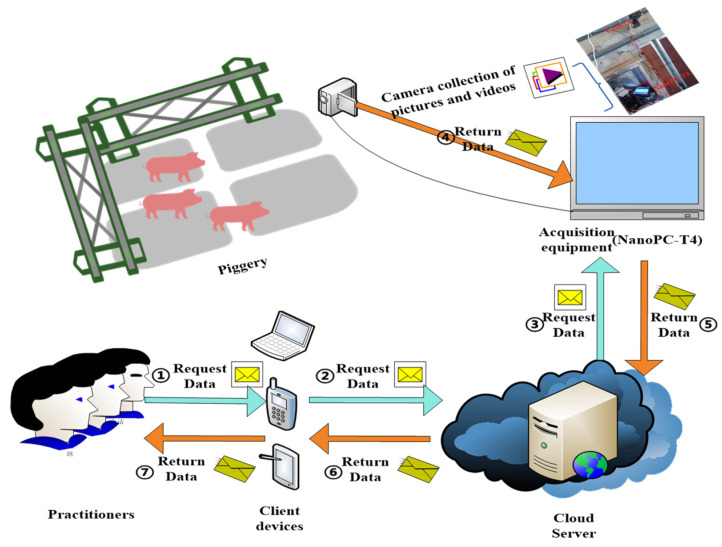
Acquisition flow chart.

This study utilized samples from pigs at the Jinghuimeng pig farm in Mengcheng, Anhui Province, China. The researchers employed a Logitech C920Pro camera (Shanghai, China, Logitech (China) Technology Co.) to capture these samples. In order to collect individual information on live pigs more efficiently, the researchers established a system that enabled remote control of the collection device. The acquisition process could be rotated remotely through the control system, which facilitates time-sharing collection under appropriate lighting conditions, thereby obtaining individual information with various characteristics. It is shown in Figure 1 below.

To obtain images and videos of live pigs, industry professionals first send a data request command to a cloud server from their client device (such as a computer). The cloud server then sends the data request command to the acquisition equipment (NanoPC-T4 (Guangdong, China, Guangzhou Yongfei Information Technology Co.)). The acquisition equipment operates the cameras to obtain images and video of the live pigs in the pig farm and returns the data to the cloud server. Finally, the cloud server sends the data back to the client device, allowing industry professionals to obtain the desired images and videos. The resulting images have a resolution of 1920 × 1080 pixels.

#### 2.1.2. Data Pre-Processing and Enhancement

To avoid small sample distinctions caused by inactive pig behaviors such as lying prone, sleeping, and lying on their stomachs, the collection threshold of the equipment is set to 5 s; simultaneously, the sample images are filtered using SSIM [26].

SSIM is a standard used to measure the quality of an image and evaluate the similarity between two images in terms of their brightness, contrast, and structure. The calculation formula is shown in Equations (1)–(7). Expressions l(x,y), c(x,y) and s(x,y) represent the brightness comparison value, the contrast comparison value, and the structure comparison value of the two images, respectively. Terms $\mu_{x}$ and $\mu_{y}$ represent the average value of *x* and *y*, while $\sigma_{x}$ and $\sigma_{y}$ represent the standard deviation of *x* and *y*. The term $\sigma_{xy}$ is represented by covariance of *x* and *y*. *N* represents the number of pixels in the image, and *C*_1_, *C*_2_, and *C*_3_ are constant values, where *C*_3_ is half of *C*_2_. The SSIM formula is shown in Equations (3)–(7) where *α*, *β*, and *γ* are all set to 1.

(1) $$l\left(x,y\right)=\frac{2\mu_{x}\mu_{y}+C_{1}}{\mu_{x}^{2}+\mu_{y}^{2}+C_{1}}$$

(2) $$c\left(x,y\right)=\frac{2\sigma_{x}\sigma_{y}+C_{2}}{\sigma_{x}^{2}+\sigma_{y}^{2}+C_{2}}$$

(3) $$s\left(x,y\right)=\frac{\sigma_{xy}+C_{3}}{\sigma_{x}\sigma_{y}+C_{3}}$$

(4) $$\mu_{x}=\frac{1}{N}\sum_{1}^{N}x_{i}$$

(5) $$\sigma_{x}={\left(\frac{1}{N-1}\sum_{i=1}^{N}{\left(x_{i}-\mu_{x}\right)}^{2}\right)}^{\frac{1}{2}}$$

(6) $$\sigma_{xy}=\frac{1}{N-1}\sum_{i=1}^{N}\left(x_{i}-\mu_{x}\right)\left(y_{i}-\mu_{y}\right)$$

(7) $$SSIM\left(x,y\right)={\left[l\left(x,y\right)\right]}^{\alpha }\cdot {\left[c\left(x,y\right)\right]}^{\beta }\cdot {\left[s\left(x,y\right)\right]}^{\gamma }=\frac{\left(2\mu_{x}\mu_{y}+C_{1}\right)\left(2\sigma_{xy}+C_{2}\right)}{\left(\mu_{x}^{2}+\mu_{y}^{2}+C_{1}\right)\left(\sigma_{x}^{2}+\sigma_{y}^{2}+C_{2}\right)}$$

The *SSIM* filtering criterion for sample images is that the higher the *SSIM* value, the smaller the dissimilarity between the two images, and the more frequently the sample image is copied. If the *SSIM* value of both sample images is greater than the threshold, it indicates that the two sampled images are repeated higher, retaining only one sample image; otherwise, all are saved.

**Figure 2:**
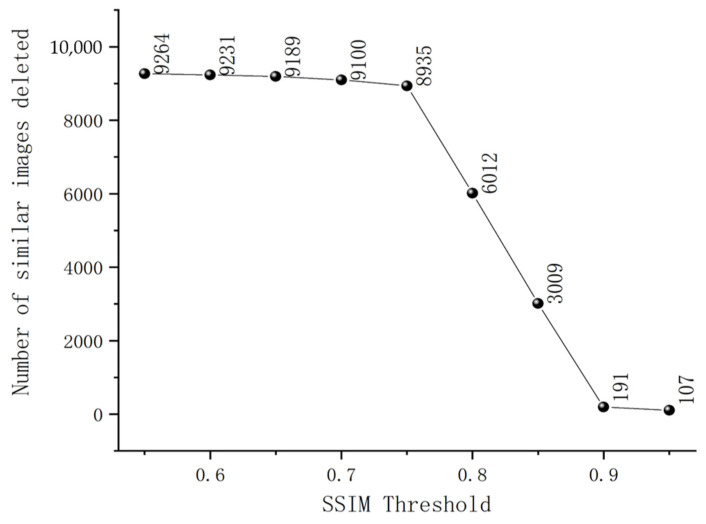
The quantity of similar images removed at different SSIM thresholds.

Originally, the dataset contained 12,215 images. As can be observed from Figure 2, the curve representing the removal of highly similar images remains relatively stable within the SSIM threshold range of 0.55–0.75 and 0.9–0.95. However, there is a noticeable drop in the curve within the 0.75–0.9 SSIM threshold range. This indicates that images within this threshold range exhibit a significant degree of similarity, with the maximum number of similar images being eliminated at an SSIM threshold of 0.75. Therefore, this study chose an SSIM threshold of 0.75. By applying this initial SSIM-based screening, a total of 3280 usable images were obtained.

**Figure 3:**
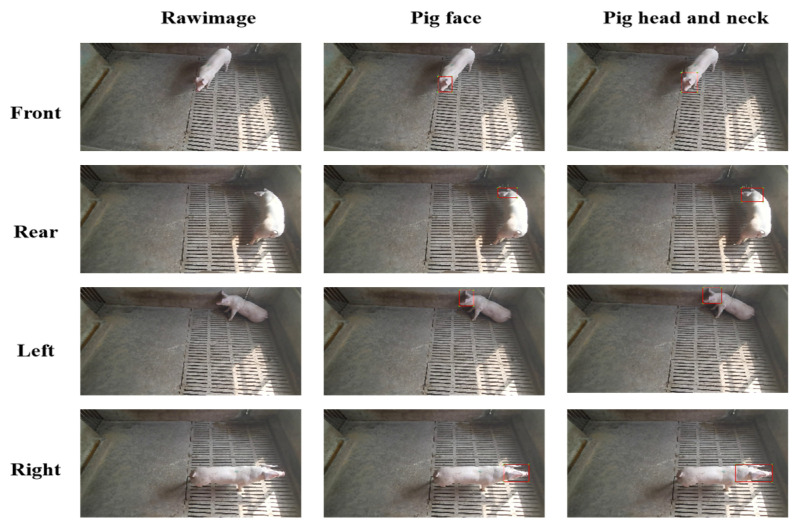
Example of annotated images from different angles made for collecting data of faces and necks of live pigs.

To ensure the accuracy of the dataset, the authors of this article liaised with the breeder to confirm that multiple captive breeding is more aligned with the farm’s economic benefits. Hence, this study focused on collecting samples from multiple captive breeding. The dataset used in this paper comprises images of one, three, and five pigs in captivity, each photographed from various angles such as front, rear, left, and right, with each pig having no fewer than 500 pictures. To develop a more effective method for individual pig recognition, this dataset includes both the pig’s face and head and neck datasets, with labeling (Windows version with Python 3.8, version 1.5.0, Heartex, San Francisco, CA, USA) software used for data labeling. Figure 3 illustrates an individual pig labeling map with a single pig as an example.

**Figure 4:**
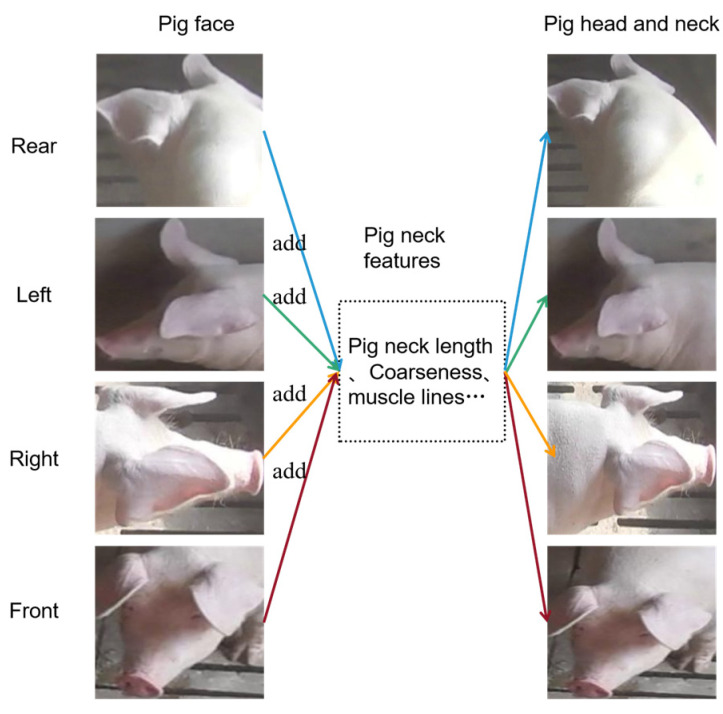
Example of comparisons between the features of faces and necks of live pigs.

As depicted in Figure 4, the head and neck of a live pig can offer valuable neck-related information derived from the pig’s face, such as its neck’s length, thickness, and muscle lines. When identifying an individual pig from its rear, left, or right profile, its facial features provide minimal or no information, making neck features a crucial aspect in identification. Furthermore, when identifying a pig from the front, the neck feature information significantly enhances the algorithm’s robustness by supplementing the rich facial features, which ultimately reduces the false detection rate.

**Figure 5:**
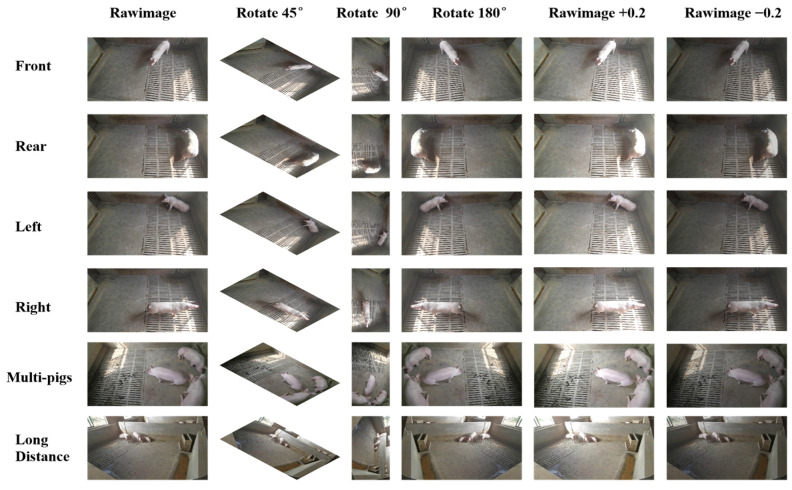
Data enhancement legend.

After filtering and integration, a total of 3280 valid images were collected from 9 pig categories, including pig1, pig2, pig3, and pig9, with front, back, left, and right views for each category. Data augmentation techniques were then applied, such as rotating clockwise by 45 and 90 degrees, flipping 180 degrees, and adjusting brightness by 0.2 times. These techniques increased the experimental sample size to six times the original amount, resulting in 19,680 valid images, as shown in Figure 5. The use of data augmentation improved the algorithm’s ability to detect small targets and provided additional information on individual pig characteristics. Finally, the experimental samples were divided into training and test sets using an 8:2 ratio.

**Figure 6:**
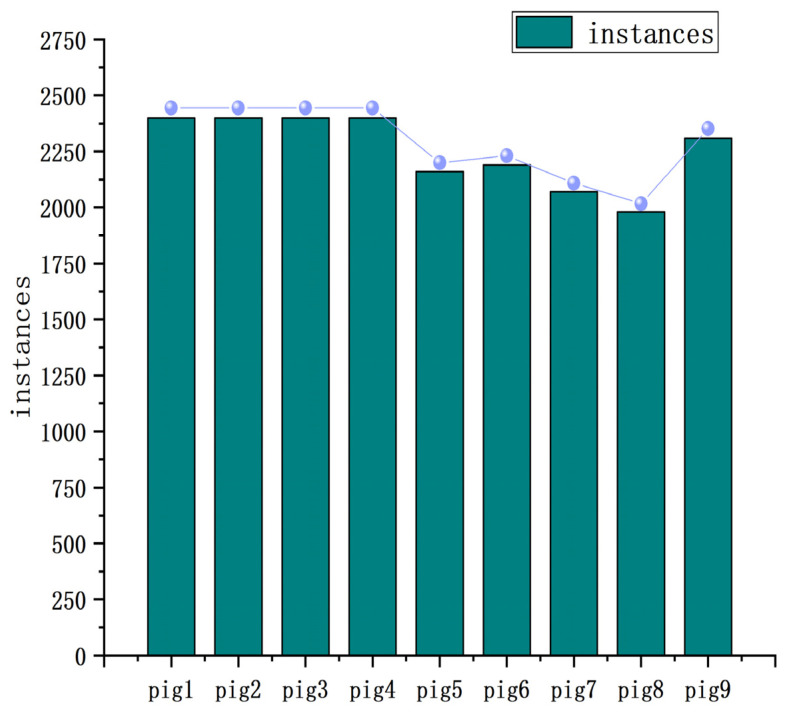
Distribution map of examples of pig categories.

Distribution of instances for each class within the training dataset is a critical component of algorithm training. Figure 6 shows the distribution of pig categories in the training data. The bar chart clearly shows that there are 2400 instances available for pig1–pig4, while pig5, pig6, pig7, pig8, and pig9 each have 2160, 2190, 2070, 1980, and 2310 instances, respectively. In addition, the graph shows that the number of instances attributed to each pig remains relatively stable, indicating a competent and well-organized distribution of data within the dataset. This robust and organized distribution of data provides the necessary support for the algorithm to effectively learn individualized pig information.

### 2.2. The Principle of the YOLOv5 Algorithm

YOLOv5 is a novel algorithm that enhances the success of the previous YOLO versions [27,28,29,30]. Compared to YOLOv3 [29] and YOLOv4 [30], YOLOv5 outperforms in weight files, inference time, and training time. The official implementation of YOLOv5 consists of four target-detection network models: YOLOv5s, YOLOv5m, YOLOv5l, and YOLOv5x. All of these models are deeper and broader than YOLOv5s. For the purpose of individual pig recognition in this project, we opted for the lightweight YOLOv5s network.

The input module utilizes three methods to handle the input images: mosaic data augmentation, adaptive anchor calculation, and adaptive image resizing. These methods guarantee uniform treatment of the input images within the algorithm.

The primary role of the backbone is to extract features using modules such as the focus module, bottleneck CSP, and spatial pyramid pooling (SPP [31]). The focus module periodically captures pixel samples from the input image and reconstructs them into a lower resolution image, effectively expanding the receptive field of each point and minimizing the loss of original information. The bottleneckCSP (Bottleneck and CSP) module effectively minimizes computation and enhances speed. The SPP [31] module employs maximum pooling with levels of 5, 9, 13, and concatenative fusion to expand the receptive field.

The neck section of the YOLOv5 algorithm uses PANET [32] based on Mask R-CNN and FPN frameworks [33,34,35] to enhance information propagation and retain spatial information, which helps to accurately locate pixels and form a mask.

**Figure 7:**
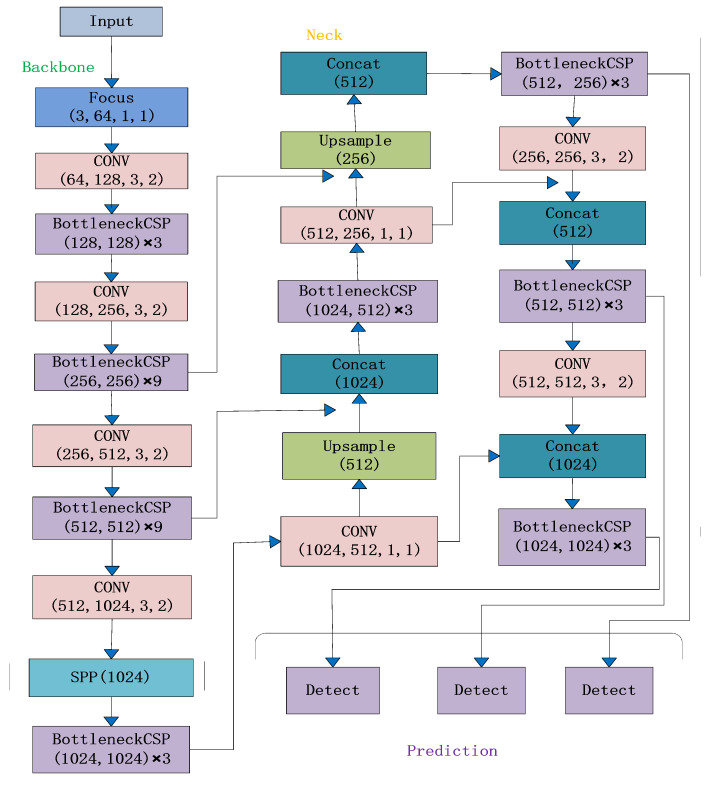
YOLOv5 network structure.

Prediction is the main detection part, where anchor boxes are applied to the feature map to generate classification probabilities, confidence levels, and the final vector of the target anchor boxes. Figure 7 shows the complete YOLOv5 algorithm.

### 2.3. Algorithm Improvement Methods

**Figure 8:**
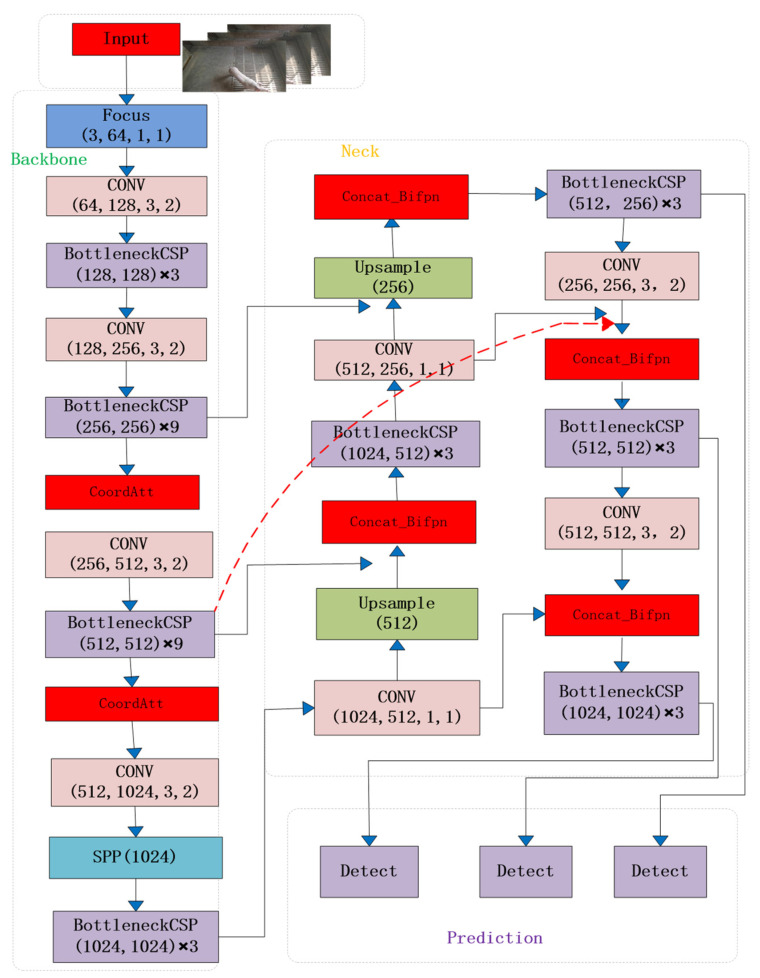
YOLOv5-KCB network structure.

To address the challenges of misdetection of individual pigs in single, multiple, dense, and long-distance pig confinement, a new YOLOv5-KCB algorithm is proposed in this paper with the network structure diagram shown in Figure 8 below, which has the following three improvements over the original algorithm:

(1) The K-means clustering distance is replaced by 1-IOU, which enhances the model’s target anchor frame adaptability.

(2) A CA attention mechanism is integrated into the backbone feature extraction network to effectively learn small target features and target location features, thereby improving the network’s feature extraction capability and robustness.

(3) The BiFPN structure is introduced for cross-layer feature fusion, which makes strategic use of information from both higher and lower layers. This expansion of the algorithm’s perceptual field enhances multiscale learning of multi-disturbance pig individuals.

#### 2.3.1. Optimizing the Target Anchor Frame

The dimensions of previous anchor boxes have a significant impact on target detection, and selecting the appropriate anchor boxes can enhance the detection accuracy of learned detectors. There are two primary methods for determining prior anchor boxes: empirical determination and clustering determination. YOLOv5 uses K-means clustering [36] to determine prior anchor boxes. In this paper, the Euclidean distance of the K-means algorithm is replaced by 1-IOU (intersection over union) (e.g., boxes, anchors) as the method for determining prior anchor boxes.

#### 2.3.2. Introducing the Coordinate Attention Mechanism

**Figure 9:**
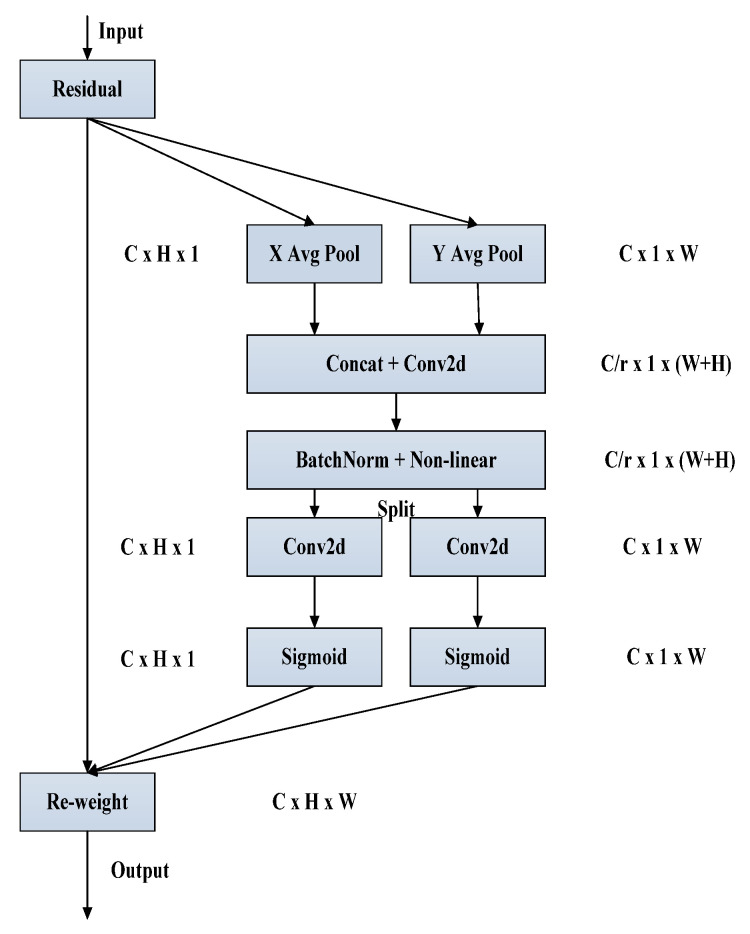
Coordinate attention mechanism.

To identify the most critical feature information for a given task from the input image data, attention mechanisms are employed. These mechanisms can be broadly classified into three aspects: spatial attention, channel attention, and self-attention. While attention mechanisms are known to significantly enhance the performance of deep neural networks, they can also pose a computational challenge for smaller mobile networks. To solve this problem, this paper experiments with SE [37] (squeeze-and-excitation), CBAM [38] (convolutional block attention module), and CA (coordinate attention) mechanisms [39], ultimately selecting the novel and efficient CA attention mechanism. CA uses precise location information to encode channel relationships and long-term dependencies while maintaining high efficiency and requiring minimal additional computation. Figure 9 illustrates the specific process.

To improve the accuracy of feature information captured by the attention module, we modified the traditional global pooling method by decomposing it into two one-dimensional feature encodings. To achieve this, input *X* undergoes an average pooling operation across horizontal and vertical dimensions, with dimensions of (*H*, 1) and (1, *W*), respectively. The equations below can be used to express the output of channel *C*, which has a height of *h* and a width of *w*.

(8) $$Z_{{}_{c}}^{h}(h)=\frac{1}{W}\sum_{0\leq i<W}x_{c}(h,i)$$

(9) $$Z_{{}_{c}}^{w}(w)=\frac{1}{H}\sum_{0\leq j<H}x_{c}(j,w)$$

By aggregating features along two spatial directions, the two transformations facilitate the production of two direction-aware feature maps. This approach allows the attention module to effectively capture long-term dependencies in one spatial direction while retaining precise location information in the other. As a result, the network can accurately identify the target of interest while excluding any interference from the background of the image.

After the information embedding is transformed, the height and width $z^{h},z^{w}$ are stitched together through a subsequent operation. The resulting feature-mapping generates spatial information in both vertical and horizontal directions through a 1 × 1 $F_{1}$ convolution operation, as illustrated in the following equation.

(10) $$f=\delta (F_{1}([z^{h},z^{w}]))$$

Then, along the spatial information, *f* is converted into tensors $f^{h}\in R^{C/r\times H}$ and tensors $f^{w}\in R^{C/r\times W}$ using a reduction rate *r*, which controls the sample size. Convolutional transformations are performed on *F_h_* and *F_w_*, respectively. Tensors are converted into tensors $f^{{}_{h}}$ and $f^{w}$, which possess an identical number of channels, as denoted by the subsequent equation:

(11) $$g^{h}=\varphi (F_{h}(f^{h}))$$

(12) $$g^{w}=\varphi (F_{w}(f^{w}))$$

The equation φ presented above represents the sigmoid activation function. Additionally, reducing the number of channels f by a suitable reduction ratio r can significantly decrease the computational effort and the complexity of the model. The resulting sums $g^{h}$ and $g^{w}$ are expanded to function as attention weights, and the resulting equation is used as the output.

(13) $$y_{c}(i,j)=x_{c}(i,j)\times g_{c}^{h}(i)\times g_{c}^{w}(j)$$

**Figure 10:**
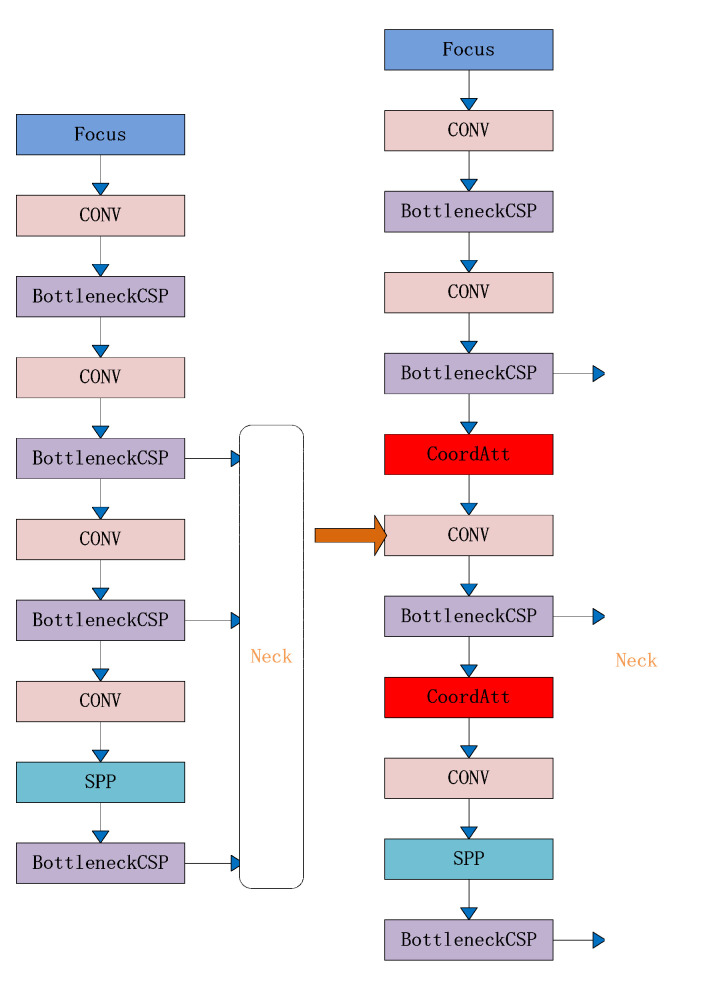
Introduction of the CA attention module.

The CA attention mechanism location is introduced, as shown in Figure 10.

#### 2.3.3. Improved Feature Fusion

**Figure 11:**
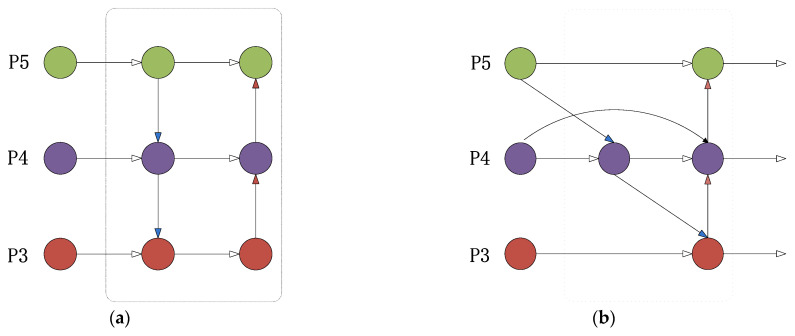
Feature-fusion graph of PANET (**a**) and BiFPN (**b**).

After extracting features from the backbone network, the utilization of both high-level and low-level features is essential for enhancing the algorithm’s performance in detecting individual pigs. Yolov5 employs PANET’s bidirectional feature fusion technique (as illustrated in Figure 11a) to combine features, which facilitates the integration and utilization of features. However, it fails to target significant feature contributions and imposes significant parameter and computational costs.

**Figure 12:**
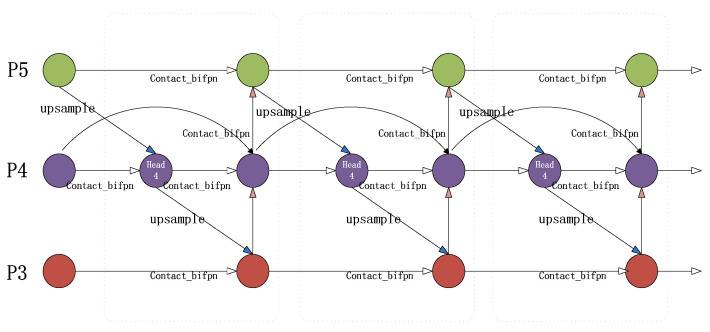
BiFPN feature-fusion module process.

**Figure 13:**
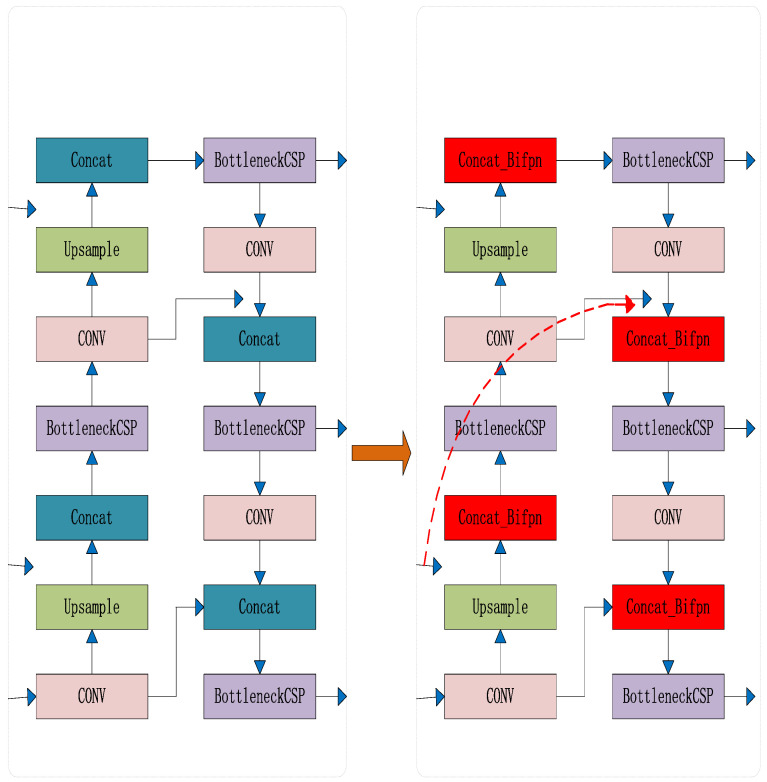
Improved BiFPN feature fusion module.

To tackle this challenge, the article introduces several techniques for experimentation, namely CARAFE [40] (a lightweight general up-sampling operator), ASFF [41] (adaptive spatial feature fusion), and BiFPN [42] (bidirectional feature pyramid network), ultimately selecting BiFPN for further use (as demonstrated in Figure 11b). The feature network pruning and fusion process is illustrated in detail in Figure 12. Initially, P3 and P5 nodes with insignificant feature contributions in the fusion feature network are removed, including nodes with only one input edge and those with no feature fusion. Subsequently, additional edges are introduced from the original input to the output nodes in P4 to integrate more features while maintaining efficiency. Finally, a pair of paths is treated as a single feature layer and repeated several times to achieve more advanced feature fusion. Because different input features have different resolutions and semantic information, which each contribute differently, the article adds additional weights through fast normalized fusion (as shown in Equation (14)) to allow the network to learn the importance of each feature layer. This improves the algorithm’s performance in individual pig detection. The network diagrams before and after the BiFPN feature fusion improvement are shown in Figure 13. In the diagram, the red module indicates the use of the BiFPN structure instead of the PANET structure for feature fusion. The red dashed arrow represents the inter-level connections within the BiFPN structure.

(14) $$O=\sum_{i}\frac{w_{i}}{\in +\sum_{j}w_{j}}\cdot I_{i}$$

In the above equation $w_{i}$ ≥ 0 is ensured by applying Relu after each $w_{i}$, and ∈ = 0.0001 is a small value to avoid numerical instability.

## 3. Results and Analysis

### 3.1. Experimental Environment

Due to the significant size of parameters and high level of computational complexity in most target detection algorithms, this paper utilizes GPUs, which offer much greater computational power than CPUs, to reduce training time and improve speed. The experimental setup in this study includes an Intel i7 9700F 3.0 GHz processor with 16 GB of memory and a 16 GB graphics card (NVIDIA GeForce RTX 2080Ti). The tabulated data presented in Table 1 provides specific details.

To maximize the overall goal, the algorithm was trained using the SGD optimizer with a batch size of 16, an initial learning rate of 0.001, a momentum of 0.92, and a weight-decay coefficient of 0.001. The learning rate was changed using the cosine annealing method, and the algorithm went through 100 iterations. All algorithms used in this research employed the same hyperparameters mentioned above.

### 3.2. Model Evaluation

In this article, we will use common evaluation metrics in deep learning [43,44], including recall (*R*), precision (*P*), average precision (*AP*), and mAP, which is the average of *AP* values across all categories. *TP* (true positives) is the number of correctly identified individual pigs, while *FN* (false negatives) is the number of missed individual pigs, and *FP* (false positives) is the number of falsely identified individual pigs. The formulas for these metrics are shown below:

(15) $$Precision=\frac{TP}{TP+FP}$$

(16) $$Recall=\frac{TP}{TP+FN}$$

(17) $$AP=\int_{0}^{1}P(R)dR$$

### 3.3. Experimental Comparison of Improved Target Anchors

This new approach has been shown to be feasible by the introduction of the average IOU as an evaluation metric. Average IOU represents the average maximum IOU between the previous box and the actual target box. The higher the average IOU, the better the previous frame obtained by the improved algorithm.

The experimental hardware is displayed above. The previous boxes were clustered using the K-means algorithm, utilizing both its original form (referred to as Cluster SSE) and an enhanced method (referred to as Cluster IOU).

It is evident that the original clustering algorithm performs better than the manual design method in determining the previous frame from Table 2. However, the improved clustering algorithm outperforms both the original clustering algorithm and the manual design method, with improvements of 6.1% and 2.1% in the average IOU, respectively. This demonstrates that the enhanced K-means algorithm surpasses the previously-used adaptive prior frame determination methods and enhances the detection of multi-scale pig face images in the model.

To further evaluate the performance of optimizing the target anchor box improvement for individual pig identification and detection, experiments were conducted using the same experimental setup and parameters on a homemade dataset of pig faces and live pigs’ heads and necks. Table 3 shows the details of the experiments, where “-” indicates the addition of a module in YOLOv5 (as shown in the following tables) except for the CBAM and CARAFE modules, which are represented by the last letter of their names. 

The experimental findings demonstrate that the YOLOv5-K algorithm, which enhances the target anchor box through K-means clustering, has considerably increased accuracy, recall, and average precision, as compared to the original YOLOv5 algorithm. Additionally, it supports the improved YOLOv5 algorithm in multi-scale live-pig individual identification.

In comparing the datasets of pig faces and live pig heads and necks, the data from the latter resulted in varying degrees of improvement in accuracy, recall, and average precision in both the original YOLOv5 and YOLOv5-K algorithms. This suggests that the head and neck dataset is better suited for discerning individual pigs.

### 3.4. Experimental Comparison with Attention Mechanisms

To thoroughly evaluate how attention mechanisms affect individual pig detection, we conducted experiments on our own dataset of pig faces and head–neck data. Under the same experimental conditions, we compared CA, SE, and CBAM attention mechanisms against the original YOLOv5 algorithm. 

The results in Table 4 illustrate that the YOLOv5-S, YOLOv5-M, and YOLOv5-C algorithms with the introduced attention mechanisms are all more effective than the original algorithm in terms of accuracy, recall rate, and mean precision. Therefore, this paper introduces the CA attention mechanism to enhance the algorithm’s anti-interference ability and target feature extraction, improving individual pig detection results.

In addition, the pig head–neck dataset has more individual pig feature information than the pig face dataset, which allows YOLOv5-S, YOLOv5-M, and YOLOv5-C to extract individual pig information better. This, in turn, boosts the algorithm’s accuracy, recall rate, and average accuracy, while also strengthening the robustness of its individual pig detection algorithm.

### 3.5. Experimental Comparison of Improved Feature Fusion

To gain a deeper understanding of the effectiveness of enhanced feature fusion algorithms for individual pig detection, a consistent dataset of pig faces and head–neck images was utilized under the same experimental conditions. The evaluated feature fusion algorithms included lightweight common up-sampling operator (CARAFE), adaptive spatial feature fusion (ASFF), bidirectional feature pyramid network (BiFPN), and the original YOLOv5 algorithm, which were compared and analyzed in Table 5.

In terms of algorithm optimization, YOLOv5-E with CARAFE, YOLOv5-A with ASFF, and YOLOv5-B with BiFPN feature fusion all showed improvements in accuracy, average precision, and recall compared to the original YOLOv5 algorithm. However, YOLOv5-B demonstrated significantly better performance in average precision and accuracy compared to the previous two improvements. As a result, this study utilized the YOLOv5-B approach with BiFPN feature fusion to extract high-level and low-level features from the backbone network in a biased manner, broaden the receptive field, and establish a stronger foundation for identifying individual pigs.

When comparing the pig face and pig head–neck datasets, it was found that the pig head–neck dataset is better suited for feature extraction and fusion. YOLOv5-A, YOLOv5-E, and YOLOv5-B all showed varying degrees of improvement in accuracy, recall, and average precision on this dataset. Notably, YOLOv5-B performed much better in feature fusion on the pig head–neck dataset compared to the pig face dataset, with a 1% improvement in average precision, making it more beneficial for individual pig identification.

### 3.6. Ablation Experiment

To verify the effectiveness of the proposed combination of modules to enhance individual pig identification, an ablation experiment was conducted. The results are presented in Table 6, and use the same pig face and pig head neck datasets while maintaining consistent experimental conditions. Compared with the original algorithm, the algorithm incorporating improved K-means clustering, CA coordinate attention mechanism, and BiFPN feature fusion showed varying degrees of improvement in accuracy, recall, and mean precision. This suggests that improved K-means clustering effectively improves the algorithm’s efficiency in learning target detection boxes. Moreover, CA’s long-term dependence on both location and channel relationships enhances the algorithm’s efficiency in learning position information and enhances prediction accuracy. Finally, BiFPN feature fusion simplifies nodes that contribute less to feature fusion, strengthens nodes that contribute more through weight allocation, and effectively combines low-level and high-level feature maps for optimal detection results.

In terms of algorithm optimization, the comparison in Table 6 shows that the YOLOv5-KCB used in this paper outperformed other algorithms in all aspects except recall, which was slightly lower than that of YOLOv5-C and YOLOv5-B, which incorporated CA alone, and CA and BiFPN together, respectively. In terms of average precision (mAP), the increase in performance was in the following order: adding one improved point (YOLOv5-K, YOLOv5-C, and YOLOv5-B), adding two improved points (YOLOv5-KC, YOLOv5-KB, and YOLOv5-CB), and adding three improved points (YOLOv5-KCB), with the improvement gradually increasing compared to the original YOLOv5 algorithm. This indicates that the three improvements in this paper are not only effective when used individually, but also have a positive correlation when used together. Overall, the YOLOv5-KCB with three improvements still performed best, with the highest average accuracy and precision, although the recall rate was lower than some individual algorithms. The average accuracy and precision for pig face recognition were 95.5% and 92.6%, respectively, 2.2% and 13.2% higher than those of the original YOLOv5, while for pig head–neck recognition, they were 98.4% and 95.1%, respectively, 4.8% and 13.8% higher than those of the original YOLOv5, further demonstrating the feasibility of YOLOv5-KCB.

**Figure 14:**
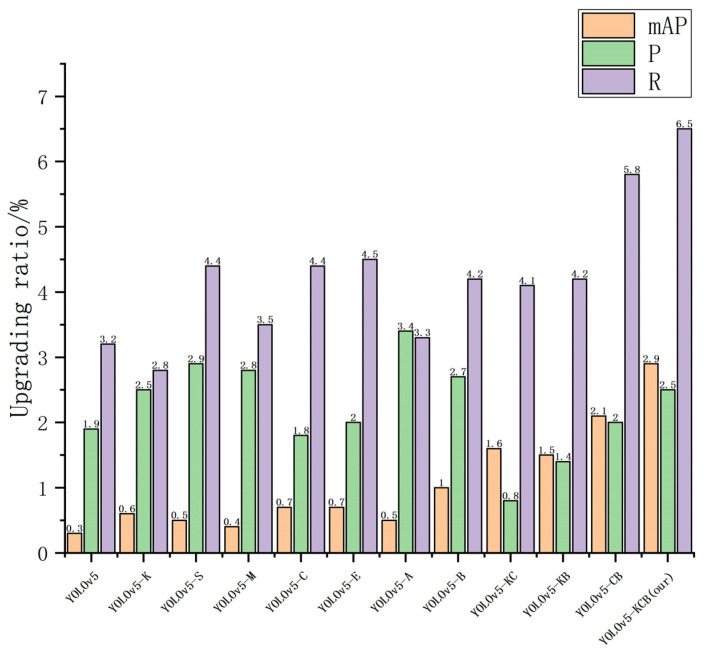
Increase in the performance of recognition of pig necks compared to pig faces.

The X-axis in Figure 14 below represents various algorithms, and the Y-axis represents the percentage improvement in pig head and neck recognition compared to pig face recognition (unit: %).

Regarding the comparison of the pig face and pig head–neck datasets, the use of the pig head–neck dataset improved the average precision, accuracy, and recall rate to varying degrees. The YOLOv5-KCB used in this paper had a lower increase in accuracy than some individual improved algorithms, but it still achieved a 2.5% increase and had the greatest improvement in average accuracy (mAP) and recall rate (R), reaching 2.9% and 6.5%, respectively, indicating that using the pig head–neck dataset for recognition is more conducive to identifying individual pigs than is using the pig face dataset.

### 3.7. Results of Ablation Experiment

**Figure 15:**
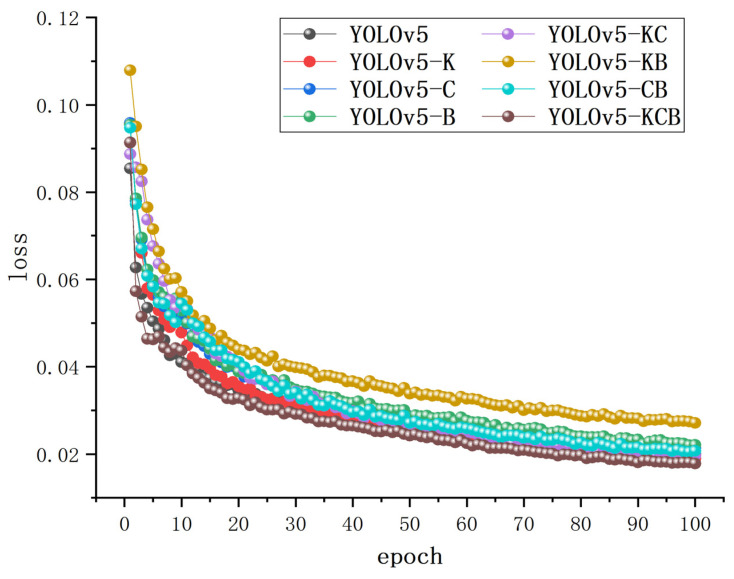
Loss curve.

The pig face, pig head and neck datasets were used for individual pig identification, with better pig head and neck identification highlighted as an example. Figure 15 illustrates the results of ablation experiments on various algorithms in terms of test-set loss. The figure shows that the value of the loss decreases rapidly in the range 0 to 15, followed by a gradual decline in the range 15 to 75. Finally, when the epoch is between 75 and 100, the loss of various algorithms drops to a stable level, and the loss value stabilizes at about 0.015–0.03 on convergence. The training process does not exhibit underfitting or overfitting.

### 3.8. Detection Results of YOLOv5-KCB

**Figure 16:**
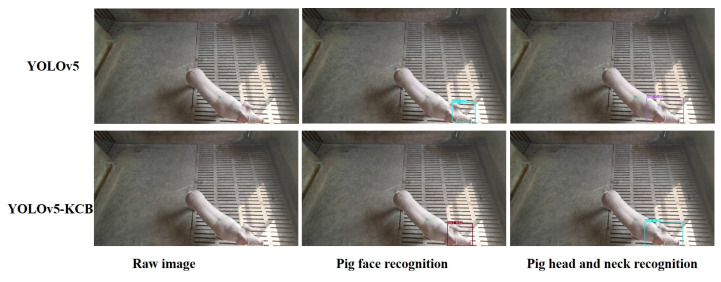
Detection of a single pig in a pen.

**Figure 17:**
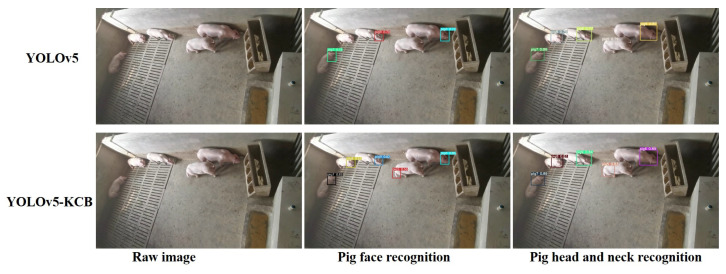
Detection of multiple pigs in a pen.

**Figure 18:**
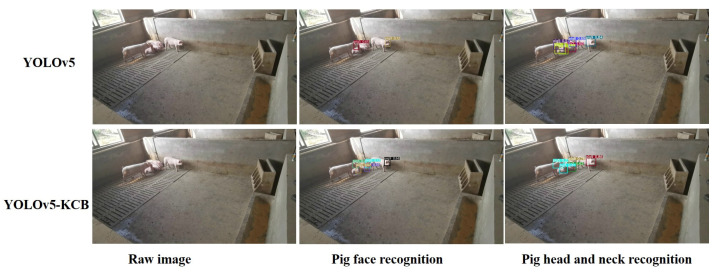
Detection of dense and distant live pigs.

In actual pig farming, there are multiple feeding environments. This study verifies the effect of YOLOv5-KCB on individual pig identification in three environments: single-headed, multi-headed, and dense and long-distance. Figure 16, Figure 17 and Figure 18 show the detection results of the original YOLOv5 algorithm and the improved YOLOv5-KCB algorithm on pig face and pig head–neck datasets.

In Figure 16, under single-pig breeding, the original image has only background interference information and no other pig obstruction or interference. Therefore, both the YOLOv5 algorithm and the YOLOv5-KCB algorithm can detect individual pigs using either the pig’s face or the pig’s head and neck. However, the classification confidence detected by the YOLOv5-KCB algorithm is higher, and the fitting effect of the detection box is more significant. In addition, the recognition effect of the pig head–neck dataset in the two datasets is better. This suggests that the YOLOv5-KCB algorithm still maintains strong robustness and accuracy under interference backgrounds and is suitable for multiple datasets.

In the case, from Figure 17, of raising multiple pigs in a pigsty, the YOLOv5 algorithm missed pig 8 under strong lighting conditions and pig 5 with few facial features, while the YOLOv5-KCB algorithm successfully detected pigs 8 and 5, but with lower classification confidence and poorer detection box alignment for pig 5. This indicates that the YOLOv5-KCB algorithm still exhibits high robustness in strong lighting conditions and situations with fewer feature points.

In the case of identifying individual pigs based on their head and neck, both the YOLOv5 algorithm and the YOLOv5-KCB algorithm detected all individual pigs, but the YOLOv5-KCB algorithm showed significant improvements in classification confidence and detection box alignment for all individual pigs. This indicates that the accuracy and robustness of the YOLOv5-KCB algorithm for identifying individual pigs in a dataset with more head and neck feature points will be further improved.

In the case, from Figure 18, of raising pigs in dense and long distance pig farms, in pig face recognition of individual pigs, the YOLOv5 algorithm missed pigs 6, 7, and 8 in dense, obstructed, and long distance small target recognition, while the YOLOv5-KCB algorithm successfully detected all individual pigs, but due to blurred facial features and obstruction of small target pigs 6 and 7, the effective facial feature information was limited and the detection boxes were offset. This indicates that the YOLOv5-KCB algorithm further enhances feature extraction of location information and fusion of high-level and low-level information. In the identification of individual pigs based on their head and neck, the YOLOv5 algorithm produced a false detection for pig 6, while the YOLOv5-KCB algorithm successfully detected pig 6. This indicates that the YOLOv5-KCB algorithm has a strong generalization ability in scenes with small, dense, and obstructed targets.

In summary: (1) Recognizing individual pigs based on their head and neck can obtain more pig features and is more beneficial in real-world scenarios than the correlative performance of pig face recognition. (2) The YOLOv5-KCB algorithm can still maintain high recognition accuracy and robustness under the differing conditions of single or multiple heads, and dense and long-distance pigs.

### 3.9. Experimental Comparison of Other Improved Algorithms

To provide a better analysis of the strengths and weaknesses of the improved algorithm presented in this article, we conducted experiments on other improved algorithms under the same dataset and experimental conditions, as shown in Table 7. Experimental results show that the YOLOv5-KCB used in this paper outperforms other algorithms in all aspects, except for slightly lower accuracy and recall rates in pig face recognition and head–neck recognition when compared to YOLOv5-M and YOLOv5-A, respectively. In particular, the YOLOv5-KCB algorithm shows a significant advantage in average accuracy, with a 2% and 4.4% improvement in pig face recognition and head–neck recognition, respectively, over the lower-performing YOLOv5-S algorithm. There was also an improvement of 1.6% and 3.8% over the highest performing YOLOv5-E. These results further confirm the feasibility of the YOLOv5-KCB algorithm proposed in this paper for individual recognition of live pigs.

### 3.10. Experimental Comparison with Other SOTA Algorithms

To further demonstrate the feasibility of the YOLOv5-KCB algorithm, a comparison experiment was conducted with the YOLOv5, YOLOX [45], and YOLOv7 [46] algorithms. The objective of the experiment was to evaluate three metrics: mAP, P, and R. The experimental results, presented in Table 8, maintained consistency in the dataset and experimental conditions to ensure reliability. 

The results demonstrate that the YOLOv5-KCB algorithm used in this study showed varying degrees of improvement across the three metrics in pig face and head–neck recognition compared to YOLOv5, YOLOX, and YOLOv7. Notably, significant improvements were observed in the YOLOv5 and YOLOX algorithms. When compared to YOLOv7, the YOLOv5-KCB algorithm showed a minor increase in accuracy, but significantly improved recall and average precision, with respective increases of 1.8% and 1% in pig head–neck recognition. These findings suggest that the YOLOv5-KCB algorithm, which incorporates optimized K-means, CA attention mechanism, and bidirectional feature pyramid networks, is highly feasible in individual pig detection.

## 4. Discussion

Traditional ear tag identification in pig farming is prone to the falling-off of tags, causing infections, and having difficulties in identification and low accuracy. More and more practitioners are turning to non-invasive identification based on deep learning algorithms, reducing manual labor in pig individual identification, and providing new possibilities for realizing welfare, systematization, and intelligence in pig production management. Wutke M et al. [47] proposed using a CNN network to detect individual pigs and provide their location and motion direction, laying the foundation for subsequent pig tracking. Chen J et al. [48] proposed a lightweight YOLOv5 network and applied it to an embedded platform for pig individual identification and early warning, achieving a detection accuracy of up to 93.5%. Wang X et al. [49] proposed a pig individual detection method based on HRNet and Swin transform, which improved the average precision by 6.8%.The above studies all used non-invasive identification and achieved certain results in pig individual identification, but did not innovate and improve specific single, multiple, small targets, dense, and occluded pig farming scenes. This paper uses the YOLOv5-KCB algorithm to optimize the target anchor box, enhance the extraction of position features, and improve the fusion of high and low-level features, improving the robustness and accuracy of pig individual identification in different scenarios. The average precision is 98.4%, which is a 4.8% improvement over the original model.

Experimental results show that the YOLOv5-KCB algorithm can accurately identify multiple pig individuals in different scenarios, with fast identification speed and high accuracy, to some extent, solving the problems of traditional pig farming ear tag identification being prone to falling off, causing infections, difficult identification, and low accuracy. In the future, combined with pig detection platforms, pig farmers can observe pigs around the clock, which is beneficial for statistics and prediction of the growth of breeding pigs, piglets, and other important pigs, and can timely deal with a series of sudden problems such as biting and climbing. In this article, the YOLOv5-KCB algorithm is highlighted as a leading comparative detection algorithm, giving rise to the expectation that it is proficient in other livestock identification beyond doubt.

However, this paper only used nine types of pig individual samples for experimentation, which has certain limitations. In order to better apply the YOLOv5-KCB algorithm in modern pig farming, the next step will be to collect more pig individual images and further improve and adjust the model to improve the practicality and accuracy of pig individual image recognition.

## 5. Conclusions

To address the challenges and limited accuracy associated with individual pig identification in pig farming, this paper presents a novel non-intrusive recognition algorithm named YOLOv5-KCB. By optimizing the adaptability of target anchor boxes, enhancing the algorithm’s feature extraction capabilities, and effectively integrating high-level and low-level information, the algorithm expands its receptiveness. Consequently, the YOLOv5-KCB algorithm achieves an average precision of 0.984 in identifying regions of pig heads and necks. Compared to the traditional manual individual pig identification method, which suffers from low efficiency, poor accuracy, and inadequate security, the non-intrusive recognition provided by the YOLOv5-KCB algorithm is both safer and more efficient, with greater accuracy. Furthermore, in comparison to existing deep learning-based individual pig identification methods, the YOLOv5-KCB algorithm demonstrates superior performance, with average precision, recall, and accuracy values of 0.984, 0.962, and 0.951, respectively. In addition, the YOLOv5-KCB algorithm has a broader recognition range, enabling convenient individual pig identification in a variety of settings such as single and multiple pig environments, as well as densely populated and long-distance pig farming environments. These findings emphasize the essential role that YOLOv5-KCB plays in monitoring pig farming and individual recognition. The introduction of YOLOv5-KCB provides a viable solution for modernizing individual pig identification technology. Future work will aim at collecting more pig head and neck data in various scenarios to validate the algorithm’s performance, increase its robustness, and establish a solid basis for intelligent recognition and management of individual pigs.

## Figures and Tables

**Table 1:** Configuration information.

Software and Hardware	Parameters
CPU	Intel i7 9700F 3.0 GHz
Opencv	3.4.1
CUDA	11.1
Cudnn	7.6.5
GPU	NVIDIAGeForce RTX2080Ti
Operating System	Windows10
Frame	Pytorch 1.7.1

**Table 2:** Comparison of average IOU (intersection over union).

Box Generation	Box Number	Avg IOU/%
Anchor Boxes	9	80.4
Cluster SSE	9	84.4
Cluster IOU	9	86.5

**Table 3:** Optimization of the performance of the target frame’s front-and-back comparison.

Networks	mAP		P		R	
	Pig Face	Pig Head and Neck	Pig Face	Pig Head and Neck	Pig Face	Pig Head and Neck
YOLOv5	0.933	0.936	0.794	0.813	0.867	0.899
YOLOv5-K	0.945	0.951	0.906	0.931	0.883	0.911

**Table 4:** Performance comparison with different attention mechanisms.

Networks	mAP		P		R	
	Pig Face	Pig Head and Neck	Pig Face	Pig Head and Neck	Pig Face	Pig Head and Neck
YOLOv5	0.933	0.936	0.794	0.813	0.867	0.899
YOLOv5-S	0.935	0.94	0.89	0.919	0.888	0.932
YOLOv5-M	0.938	0.942	0.933	0.961	0.867	0.902
YOLOv5-C	0.941	0.948	0.915	0.933	0.899	0.943

**Table 5:** Performance comparison of improved feature-fusion algorithm.

Networks	mAP		P		R	
	Pig Face	Pig Head and Neck	Pig Face	Pig Head and Neck	Pig Face	Pig Head and Neck
YOLOv5	0.933	0.936	0.794	0.813	0.867	0.899
YOLOv5-E	0.939	0.946	0.911	0.931	0.892	0.937
YOLOv5-A	0.938	0.943	0.898	0.932	0.901	0.934
YOLOv5-B	0.947	0.957	0.917	0.944	0.881	0.923

**Table 6:** Comparison table of ablation experiments.

Networks	mAP		P		R	
	Pig Face	Pig Head and Neck	Pig Face	Pig Head and Neck	Pig Face	Pig Head and Neck
YOLOv5	0.933	0.936	0.794	0.813	0.867	0.899
YOLOv5-K	0.945	0.951	0.906	0.931	0.883	0.911
YOLOv5-C	0.941	0.948	0.915	0.933	0.899	0.943
YOLOv5-B	0.947	0.957	0.917	0.944	0.881	0.923
YOLOv5-KC	0.949	0.965	0.921	0.929	0.894	0.935
YOLOv5-KB	0.948	0.963	0.922	0.936	0.886	0.928
YOLOv5-CB	0.95	0.971	0.908	0.928	0.901	0.959
YOLOv5-KCB (proposed)	0.955	0.984	0.926	0.951	0.897	0.942

**Table 7:** Performance comparison with other detection algorithms.

Networks	mAP		P		R	
	Pig Face	Pig Head and Neck	Pig Face	Pig Head and Neck	Pig Face	Pig Head and Neck
YOLOv5-S	0.935	0.94	0.89	0.919	0.888	0.932
YOLOv5-M	0.938	0.942	0.933	0.961	0.867	0.902
YOLOv5-E	0.939	0.946	0.911	0.931	0.892	0.937
YOLOv5-A	0.938	0.943	0.898	0.932	0.901	0.934
YOLOv5-KCB (proposed)	0.955	0.984	0.926	0.951	0.897	0.962

**Table 8:** Performance comparison between YOLOv5-KCB and other SOTA detection algorithms.

Networks	mAP		P		R	
	Pig Face	Pig Head and Neck	Pig Face	Pig Head and Neck	Pig Face	Pig Head and Neck
YOLOv5	0.933	0.936	0.794	0.813	0.867	0.899
YOLOX	0.938	0.942	0.901	0.931	0.892	0.938
YOLOv7	0.949	0.966	0.925	0.948	0.915	0.952
YOLOv5-KCB (proposed)	0.955	0.984	0.926	0.951	0.897	0.962

## Data Availability

Not applicable.
